# Improved high-resolution pediatric vascular imaging with Gadofosveset-Enhanced 3d respiratory navigated IR GRE imaging compared to 3D bSSFP imaging

**DOI:** 10.1186/1532-429X-17-S1-P418

**Published:** 2015-02-03

**Authors:** Animesh Tandon, James W Parks, Sassan Hashemi, Denver Sallee, Tim Slesnick

**Affiliations:** 1 School of Medicine, Emory University, Atlanta, GA, USA; 2 Children's Healthcare of Atlanta, Atlanta, GA, USA

## Background

Improved delineation of vascular structures, including coronary arteries and pulmonary veins, is a common indication for CMR imaging in children and requires high spatial resolution given small structure sizes. Currently, pre-contrast 3D, respiratory navigated, T2 prepared, fat saturated imaging with a bSSFP readout (3D bSSFP) is commonly used; however, these images can have limitations including blood pool inhomogeneity and exaggeration of metal artifact. We sought to compare imaging after administration of a blood pool contrast agent, gadofosveset trisodium (GT), with 3D, respiratory navigated, inversion recovery prepared imaging with a gradient echo readout (3D IR GRE) to the standard 3D bSSFP for imaging of pediatric vasculature.

## Methods

All patients were imaged on a 1.5 Tesla system. For both sequences, VCG triggering was used with acquisition during a quiescent period of the cardiac cycle. Shot duration, spatial resolution, and slice thickness were kept similar between the two methods. 3D bSSFP imaging was performed pre-contrast, and 3D IR GRE imaging was performed 5 minutes after administration of GT. We devised a vascular imaging quality score (VIQS, Table 1) with scores for coronary arteries, pulmonary arteries and veins, blood pool homogeneity, and metal artifact. Scoring was performed on axial reconstructions of isotropic datasets by two independent readers and differences were adjudicated. Imaging scores were compared using paired t-tests, and p<0.05 was considered significant.

## Results

**Figure 1:**
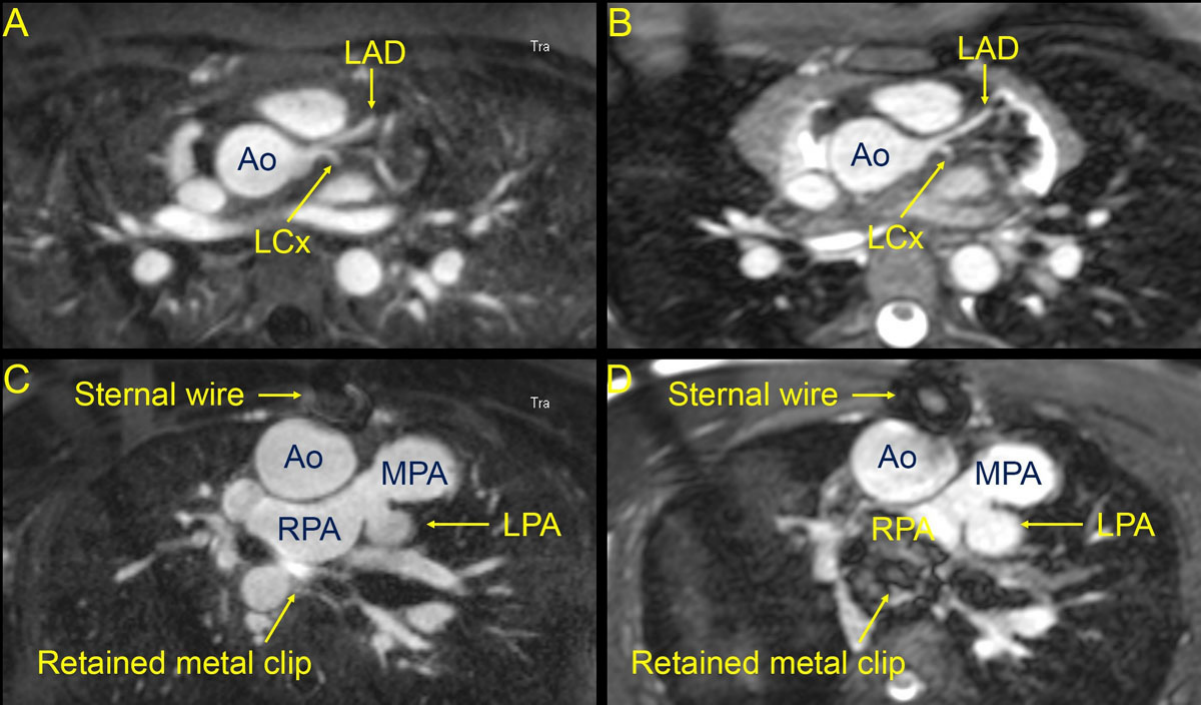
Excellent LCA imaging by both 3D IR GRE (1A) and 3D bSSFP (1B); a patient with pulmonary atresia status post RV-PA conduit with improved imaging by 3D IR GRE (1C) near a metal clip compared to 3D bSSFP (1D).

35 patients had both 3D bSSFP and 3D IR GRE imaging performed. 3D IR GRE imaging showed improved overall vascular imaging compared to 3D bSSFP for all patients when comparing non-metal VIQS scores (13.7 ± 1.7 vs. 7.8 ± 4.4, p<0.01). When analyzing patients with intrathoracic metal (n=17), 3D IR GRE again showed improved VIQS (16.9 ± 1.3 vs. 7.9 ± 4.9, p<0.01). 3D IR GRE showed significantly improved VIQS scores for pulmonary vein imaging and blood pool homogeneity (p<0.01). For RCA, LCA, pulmonary arteries, and metal artifact, the 3D IR GRE VIQS scores were higher but did not reach statistical significance (p=0.15, 0.08, 0.06, and 0.12, respectively). 3D IR GRE studies had a longer average duration (7:57 ± 4:06 minutes vs. 6:02 ± 2:26, p<0.01). There was no correlation of heart rate to VIQS for either sequence. Example images are shown in Figure 1: Excellent LCA imaging by both 3D IR GRE (1A) and 3D bSSFP (1B); a patient with pulmonary atresia status post RV-PA conduit with improved imaging by 3D IR GRE (1C) near a metal clip compared to 3D bSSFP (1D).

## Conclusions

Respiratory navigated 3D IR GRE imaging after administration of GT provides improved vascular imaging in pediatric patients compared to pre-contrast 3D bSSFP imaging, as well as improved imaging in patients with intrathoracic metal. It should be considered a viable alternative in this challenging patient population when high spatial resolution vascular imaging is needed.

## Funding

N/A.

**Table 1:** Vascular imaging quality score

	Score			
Category	0	1	2	3

Right coronary artery (RCA)	Ostium not seen	RCA ostium clear but remainder of vessel is not	RCA imaged clearly to acute margin	RCA imaged clearly to the inferior cardiac surface

Left coronary artery (LCA)	Ostium not seen	LCA ostium clear but remainder of vessel is not	LCA bifurcation clear	Left anterior descending seen along anterior interventricular groove; left circumflex seen in left posterior atrioventricular groove

Pulmonary arteries (PAs)	Branch PAs not clearly defined	Right pulmonary artery and left pulmonary artery seen	Able to identify right upper, right lower, left upper, and left lower PA	Able to identify segmental branches off of at least 2 of right upper, right lower, left upper, and left lower PA

Pulmonary veins	No vein insertion to left atrium identifiable	2 or more vein insertions clear	All 4 vein insertions clear	All 4 veins clearly seen branching

Blood-pool homogeneity	Heterogeneity in a majority of structures	Heterogeneity in multiple chambers/vessels (e.g. left atrium and left ventricle)	Heterogeneity in single chamber/vessel (e.g. left atrium)	Homogeneous blood pool in all vascular structures

Metal artifact	Non-diagnostic due to metal artifact	Large metal artifact, unable to read-through	Artifact outside the position of the metal, but able to read through	Artifact limited to metal position only

Sequence must meet criteria for lower scores to achieve higher scores. Metal artifact scores used only for patients with intrathoracic metal.

